# Teaching postsecondary students about the ethics of artificial intelligence: A scoping review protocol

**DOI:** 10.1371/journal.pone.0329020

**Published:** 2025-07-28

**Authors:** Calvin Hillis, Maushumi Bhattacharjee, Batool AlMousawi, Tarik Eltanahy, Sara Ono, Marcus Hui, Ba’ Pham, Michelle Swab, Gordon V. Cormack, Maura R. Grossman, Ebrahim Bagheri, Zack Marshall

**Affiliations:** 1 The Creative School, Toronto Metropolitan University, Toronto, Ontario, Canada; 2 Faculty of Law, McGill University, Montreal, Quebec, Canada; 3 Dalla Lana School of Public Health, University of Toronto, Ontario, Canada; 4 Department of Biology, Queens University, Kingston, Ontario, Canada; 5 Toronto Health Economics and Technology Assessment Collaborative, University of Toronto, Toronto, Ontario, Canada; 6 Health Sciences Library, Memorial University, St. John’s, Newfoundland and Labrador, Canada; 7 David R. Cheriton School of Computer Science, University of Waterloo, Waterloo, Ontario, Canada; 8 Faculty of Information, University of Toronto, Toronto, Ontario, Canada; 9 Department of Community Health Sciences, University of Calgary, Calgary, Alberta, Canada; 10 School of Social Work, McGill University, Montreal, Quebec, Canada; University of Saskatchewan, CANADA

## Abstract

The field of AI carries inherent risks such as algorithmic biases, security vulnerabilities, and ethical concerns related to privacy and data protection. Despite these risks, AI holds significant promise for social good, with applications ranging from improved healthcare diagnostics to enhanced education strategies. Teaching AI ethics in postsecondary settings has emerged as one of the strategies to mitigate AI-related harms. The objectives of this review are to (1) synthesize existing research related to teaching postsecondary students about the principles and practice of ethics and AI, and (2) identify how educators are evaluating changes in student knowledge, skills, attitudes, and behaviors. This scoping review will follow the first five steps articulated by Arksey and O’Malley. A structured search strategy developed by an academic librarian incorporates three primary concept groups related to education, AI, and ethics. Database search strategies emphasize sensitivity rather than precision, given that a supervised machine learning tool will be used to assist in the identification of relevant abstracts. Searches will be conducted in the following academic databases: PubMed, Embase, Scopus, ERIC, LISTA, IEEE Xplore, APA PsycInfo, and ProQuest Dissertations and Theses. Results will include an up-to-date synthesis of the current state of AI ethics education in postsecondary curricula, evaluated teaching strategies, and potential outcomes associated with AI ethics education. Search results will be reported according to the PRISMA-ScR checklist. Data charting will focus on AI ethics pedagogy. This review will inform future research, policy development, and teaching practices, offering valuable insights for educators, policymakers, and researchers working towards responsible AI integration. Findings will contribute to enhanced understandings of the complexities of AI ethics education and have the potential to shape the ways trainees in multiple disciplines learn about the ethical dimensions of AI in practice.

## Introduction

The field of Artificial Intelligence (AI) is booming. While the term “artificial intelligence” was first coined at the 1956 Dartmouth conference [1], the launch of ChatGPT in November 2022 catalyzed newfound interest in AI including changes in public opinion [2]. Academic and public discourse highlights the potential impacts of AI, including societal transformation, huge potential benefits, and imminent risks [3]. In Roe and Perkins’ [4 p1] recent discourse analysis of AI in UK news media headlines, “…results show that there is a complex and at times paradoxical portrayal of AI in general and ChatGPT as well as other Large Language Models”. Most recently, the Group of Seven (G7) leaders introduced the Hiroshima AI Process including International Guiding Principles and a Code of Conduct for organizations developing advanced AI systems [5]. As AI gains increasing prominence in the public consciousness, there is a growing imperative for the establishment of policies to ensure that the applications of AI systems are firmly rooted in ethical considerations.

The field of AI carries inherent risks, encompassing technical challenges such as algorithmic biases, security vulnerabilities, and ethical concerns related to privacy and data protection [6,7]. Despite these risks, AI holds significant promise for social good, with applications ranging from improved healthcare diagnostics to enhanced educational strategies [8–10]. However, ethical considerations are paramount to mitigate potential harm [11], requiring transparent and accountable algorithmic decision-making, proactive efforts to address biases in training data, and robust security measures [12]. Striking a delicate balance between realizing the transformative benefits of AI and managing its associated risks is crucial for a responsible and impactful integration of AI technologies into society.

AI ethics education is one of the proposed approaches to mitigating AI-related harms [11]. By integrating ethics into AI education, future AI practitioners across disciplines will be able to develop fair, accountable, and transparent AI systems [11]. The proposed scoping review is based on the following assumptions: (1) AI ethics content is integrated in relevant postsecondary training including computer science, engineering, business, health, social sciences, and humanities curricula, (2) strategies for teaching about AI ethics have been evaluated, and (3) teaching about AI ethics leads to better outcomes. We will explore each of these assumptions in turn.

### AI and ethics in course content

The teaching of AI ethics varies across disciplines and is informed by different paradigmatic assumptions and ideologies. This lack of standardization hinders interdisciplinary collaboration and consistency in curricula. For example, ethical philosophy and its jargon is used in Humanistic Social Science (HSS) courses as assumed knowledge. Computer Science (CS) courses often do not translate technical details, programming, and equations to be discernible to non-majors [12]. In addition, CS courses often reference industry-specific guidelines such as the Association for Computing Machinery (ACM) Code of Ethics, while HSS content focuses on the human impact of AI systems [12]. Thus, AI ethics discourse often adopts discipline-specific norms and philosophical assumptions. The “constructed distinctions” surrounding each discipline prompt inherent separation [12].

In a review of 94 international CS ethics course syllabi, researchers reflected on the ethical considerations taught to students [13]. They found that courses generally consisted of the instructor’s relativist conception of “doing good”, the traditions of CS, and the tensions that arise while navigating industry demands and societal concerns [13]. This relativist influence was also noted in a syllabi review of Australian universities’ CS ethics courses [14]. The review found no standardized macro-ethical agenda to guide AI ethics training and evaluation. Similarly in an international review, Suárez and Varona [15] analyzed 503 course syllabi across 66 universities and 16 countries. Their results align with previous findings, underlining how existing AI ethics curricula do not conform to a universal set of circulatory norms [15]. Of the 503 course syllabi examined, 84.69% had poorly articulated learning outcomes, little to know overlap, and minimal differentiation between the knowledge, values, and goals to be taught. Overall, research suggests that the existing approaches to teaching ethics of machine learning and AI lack standardized structure and cohesive goals, with no universal definition of “ethics” [12,16,17]. It is important to note that this variation is not necessarily counter-productive to AI ethics discourse but rather reflects the diverse value systems from around the world that AI ethics discourse can draw from to better serve diverse populations.

### Strategies for teaching about ethics and AI

Javed and colleagues [16] used a method called Latent Dirichlet Allocation (LDA) to analyze how AI ethics is taught in university courses. They looked at 166 courses from different parts of the world, focusing on who teaches them, where they are taught, and at what cognitive level [16]. Their analysis identified the ways AI ethics is taught from different technical, legal, and value-based perspectives depending on field of study [16]. In a separate study about strategies for teaching AI ethics, Raji, Scheuerman, and Amironesei [12] observed a rigid adherence to specific methodologies, influenced by disciplinary preferences, contributing to a separation between the HSS, which tends to favor qualitative methods, and STEM disciplines that value quantitative and computational approaches. The lack of efforts to bridge this gap seems to worsen the division. In HSS courses, there is an assumption of prior knowledge of ethical philosophy, while CS courses struggle to present technical details in a way that is accessible to non-majors. This divergence in the discourse on AI ethics, shaped by industry-specific guidelines in CS and a focus on human impact in HSS, influences and is reflected in approaches to teaching about ethics and AI.

Pedagogical strategies employed in CS ethics education may point to effective approaches to teaching AI ethics. Simulations and role-playing have been shown to be effective in CS ethics instruction [17,18]. Simulations reflecting real-life situations enhance students’ ethical capacities and interest in CS ethics through technical courses [11,13,17,18]. Role-playing activities prompt students to translate ethical decisions into code, assess biases, and experience decision-making from various perspectives in algorithmic system development, emphasizing the importance of diverse considerations [18]. The integration of role-playing into “in situ” approaches, exemplified by courses like “Deep-tech ethics” [19], significantly enhances students’ conceptual understanding and engagement with ethics. There are instances of these pedagogical strategies being applied in AI ethics contexts, though a scoping review that captures these educational trends has yet to be carried out.

### Outcomes of teaching about ethics and AI

This review aims to consolidate existing knowledge on teaching postsecondary students the principles and application of ethics within the development and utilization of AI. While some studies have presented learning outcomes and discussed assessment methods, there is a noticeable lack of comprehensive information on this topic [20,21]. Scholars have shared insights on the desired learning outcomes for university students in AI ethics courses. These encompass heightened awareness of ethical considerations in AI development, fostering collaborative understanding among students from diverse backgrounds, refining critical assessment skills for ethically informed decisions in AI, applying ethical theories to AI-related dilemmas, recognizing algorithmic biases, incorporating ethical considerations into AI system design, showcasing ongoing interest in AI ethics beyond the course, and more [11,22–24]. Evaluating these outcomes may involve a blend of quantitative measures (e.g., surveys, exams) and qualitative assessments (e.g., class discussions, projects) [18,24]. Incorporating real-world case studies can offer a practical evaluation of students’ proficiency in applying AI ethics principles in intricate situations [8,24,25]. Some scholars critique the impact of AI ethics principles, noting their often-ineffectual nature in practice, failing to address the societal damages of AI technologies [26]. This gap between principles and technological reality poses risks, diverting resources away from potentially more impactful endeavors [26]. To address these challenges, Munn suggests exploring alternative approaches to AI justice that extend beyond ethical principles, encompassing a broader consideration of oppressive systems and a focused examination of accuracy and auditing [26].

## Materials and methods

Despite the volume of research in this field, this scoping review is the first to document what is known about teaching postsecondary students about the ethics of AI, with particular attention to course content, teaching strategies, identified learning outcomes, and approaches to evaluating student learning. Drawing on qualitative, quantitative, and mixed methods research, this review will be conducted in accordance with the scoping review methods articulated by Arksey and O’Malley [27] including the first five steps: (1) identifying the research questions; (2) identifying relevant studies; (3) study selection; (4) charting the data; and (5) collating, summarizing, and reporting the results. A Preferred Reporting Items for Systematic Review and Meta-Analysis Protocols (PRISMA-P) checklist (Supporting Information) [28], as well as a Preferred Reporting Items for Systematic reviews and Meta-Analyses extension for Scoping Reviews (PRISMA-ScR) checklist (Supporting Information) [29] has been completed,

### Timeline

This scoping review is currently ongoing. The initial database search has been completed, and we are preparing to begin the screening and data charting phases. We anticipate that screening and data charting will take approximately 1–2 months to complete. Following that, we expect to spend an additional month on collating results and writing the final report,

### Step 1: Identifying the research questions,

The objectives of this review are to: (1). Synthesize existing research related to teaching postsecondary students about principles and practice of ethics in the development and use of AI, with particular attention to fairness, accountability, transparency, bias, and explainability.

(2) Identify how educators are evaluating changes in student knowledge, skills, attitudes, and behaviors consistent with evolving social expectations and values towards AI.

Two research questions have been identified:

(1) What is known about teaching postsecondary students about the principles and practice of ethics in the development and use of AI?

(2) How can students gain the knowledge, skills, attitudes, and behaviors consistent with evolving social expectations and values towards AI?

### Key concepts

This review focuses on teaching ethics to postsecondary students for the ethical use and development of AI. Each concept is defined below.

Artificial Intelligence. The term “artificial intelligence” has evolved tremendously since its conception, moving from Arthur Lee Samuel’s simple checkers program to complex transformer neural networks such as Open AI’s Chat GPT [4]. For purposes of this review, AI includes reference to artificial intelligence, computing, deep learning, computing algorithms, machine learning, natural language processing, and language learning.

Ethics. The concept of AI ethics encompasses the ethical considerations and principles guiding the development, deployment, and use of AI systems. Several guidelines have been proposed to address AI ethics [30], with attention to key dimensions such as accountability, algorithmic bias, fairness, transparency, and explainability [31]. Each concept is defined below.

Accountability in AI ethics refers to the responsibility and answerability of individuals, organizations, and systems for the impact of AI technologies on society [32]. It involves defining clear lines of responsibility for the development, deployment, and consequences of AI systems. Frameworks such as the IEEE Global Initiative on Ethics of Autonomous and Intelligent Systems [33] emphasize the importance of accountability in AI decision-making processes.

Algorithmic bias refers to the unintentional and discriminatory outcomes produced by algorithms and data-driven decision-making systems [34]. This bias may arise from historical discrimination embedded in data sources, resulting in the inadvertent amplification of existing biases. Even when sensitive attributes are omitted, correlations within the data can lead to biased algorithmic behavior through proxy variables. Addressing algorithmic bias requires a proactive and discrimination-conscious approach in the design and implementation of data mining systems.

Algorithmic fairness encompasses metrics, methods, and representations aimed at ensuring equitable and unbiased outcomes in algorithmic decision-making [34, 35]. This field emerges from the growing influence of algorithms in diverse domains and considers the socio-technical impacts of bias and discrimination [36]. Algorithmic fairness extends beyond traditional technical domains, integrating insights from social sciences, law, economics, philosophy, sociology, political science, and communication. The goal of algorithmic fairness is to create decision-making systems that consider ethical and societal implications, reducing bias and increasing equity in their outputs.

Transparency in AI ethics pertains to the openness and comprehensibility of AI systems. Transparent AI systems enable users, stakeholders, and the public to understand how decisions are made [37]. Explainability involves the ability to understand and interpret how AI systems arrive at specific decisions or predictions [31, 37]. Explainable AI (XAI) serves as a pivotal subset of AI, aiming to demystify the functioning of complex models and algorithms [37]. The primary objective of XAI is to bring transparency to the decision-making processes of various machine learning algorithms [37].

Postsecondary Education. For the context of this review, postsecondary education includes college and university settings, including diploma, undergraduate, and graduate training.

### Step 2: Identifying relevant studies

The structured search strategy for this review was developed by an academic librarian in consultation with the review team. Based on the concepts identified above, the search incorporates three primary concept groups that include both keyword terms applied to database fields such as titles and abstracts, as well as controlled vocabulary terms where applicable. The first concept group consists of words relating to education, such as “teaching”, “universities”, and “postsecondary”. The second concept group includes words relating to ethics, and the third concept group relates to AI. While there are many synonyms and related words for AI, terms such as “machine learning” or “artificial intelligence” are typically used in the article description or abstract. The three concept groups were combined using Boolean AND. Database search strategies emphasize sensitivity rather than precision [38], given that a supervised machine learning tool [39] will be used to assist in the identification of relevant abstracts.

An initial limited search of PubMed and Scopus was undertaken to identify articles on the topic. The text words contained in the titles and abstracts of relevant articles, and the index terms used to describe the articles were used in the development of a sample search strategy for PubMed (S1 Table). The initial search strategy, including all identified keywords and index terms, has been adapted for each included database. No language limiters will be imposed during the database searches. In addition, publication date limiters will not be used as the discussion of ethics in AI has been ongoing for several decades.

Sources to be searched include: PubMed (Medline), Embase (via embase.com), Scopus, ERIC (via EBSCOhost), LISTA (via EBSCOhost), IEEE Xplore, APA PsycInfo (via EBSCOhost), and ProQuest Dissertations and Theses.

### Step 3: Study selection

All identified citations will be collated and uploaded into the Continuous Active Learning® tool (CAL®), which uses supervised machine learning to rank titles and abstracts based on relevance to the search [39]. CAL® is a machine-learning tool that continuously re-ranks the abstracts based on screening decisions, so that the abstracts that are most likely to meet the inclusion criteria are presented before those that are less likely [39]. Screening ends when the remaining abstracts are very unlikely to meet the inclusion criteria. CAL® has been demonstrated to have comparable or superior accuracy to that which would have been achieved by exhaustive manual screening of every retrieved abstract, with much less human effort [39]. Because CAL® presents for screening only the abstracts that are reasonably likely to meet the criteria, it is possible to employ broad searches that retrieve more abstracts than could reasonably be screened, and there is less need to deduplicate the search results from multiple databases prior to screening.

After a pilot testing phase, each reference will be screened by a team of two independent reviewers for assessment against the review inclusion criteria. Reviewers will manually keep track of their decisions. Once they have both screened five consecutive sets of 100 references in which the inclusion rate is below 5%, screening on title and abstract will stop, as recommended by the CAL® developers. Screening results will then be extracted from the CAL® system and imported into EPPI-Reviewer [40]. This will include four separate RIS files: Reviewer (1) Includes, Reviewer (1) Excludes, Reviewer (2) Includes, Reviewer (2) Excludes. Within EPPI-Reviewer, using a Boolean search, any differences between Reviewer (1) and (2) will be identified and reviewed by a third member of the team.

### Study selection criteria: screening on title and abstract

The inclusion criteria for screening on title and abstract are:

(1) Language: Written in English. Due to team capacity only references published in English will be included in the review. Any non-English references will be identified in a separate category and the number of references in this group will be clearly reported.
(2) Setting: Postsecondary education (college or university)
(3) Topic: AI AND ethics

The exclusion criteria for screening on title and abstract are:

(1) Language: The article is not written in English
(2) Setting: Primary or secondary school (K-12), or non-educational setting such as industry
(3) Topic: Not related to AI OR not related to ethics

### Study selection criteria: screening on full text

Sources included after screening on title and abstract will be retrieved and screened on full text in EPPI-Reviewer. The full article will be assessed in detail against the inclusion criteria by two or more independent reviewers. Reasons for exclusion of sources of evidence at full text will be recorded and reported in the scoping review. Any disagreements that arise between the reviewers at each stage of the screening process will be resolved through discussion, or with an additional reviewer. The results of the search and the screening process will be reported in full in the final scoping review and presented using a PRISMA flow diagram.

The inclusion criteria for screening on full text are:

(1) Language: Written in English
(2) Setting: Postsecondary education (college or university)
(3) Topic: Ethical AI pedagogy/curriculum
(4) Focus: Ethical AI pedagogy/curriculum is the primary focus of the paper

The exclusion criteria for screening on full text are:

(1) Language: The article is not written in English
(2) Setting: Primary or secondary school (K-12), or non-educational setting such as industry
(3) Topic: Not related to AI OR not related to ethics
(4) Format: Conference/ tutorial workshop (outside of postsecondary environment)
(6) Format: Rationale/ argument for teaching about ethical AI (why this is needed)
(7) Focus: Student diversity/inclusion in AI education
(8) Focus: Student perspectives on ethical AI

While important, articles that are excluded due to Format do not provide the specific topic insight that we are seeking in this review. An example of a reference coded as “AI Ethics education review” is “More than ‘if time allows’: the role of ethics in AI education” [20]. This article analyzes a selection of AI ethics and technical AI courses to understand which ethics related topics instructors cover in their courses. Similarly, “The need for health AI ethics in medical school education” [41] would be coded as “Rationale/ arguments for teaching about ethical AI”. This article notes a technological shift in the medical field, highlights the potential of AI in healthcare, and provides an argument for introducing AI ethics instruction in medical school. While useful for the discipline, papers such as these typically review AI course syllabi, or emphasize the importance of teaching AI ethics, while our review is primarily focused on collecting data from the peer-reviewed literature about pedagogical approaches to AI ethics instruction.

### Step 4: Charting the data

Data charting will be done manually by one team member and verified independently by a second team member. If the second reviewer has questions or concerns, the two team members will work together to determine eligibility and inclusion for data synthesis and charting.

### Data-charting elements

The following information will be charted from each included study: (1) article format; (2) curriculum/course content; (3) educational setting; (4) educational strategies; (5) learning outcomes evaluation method; (6) learning outcomes analysis method; (7) reported learning outcomes; and (8) outcomes direction of change.

Article Format. Reviewers will indicate relevant elements related to course format including: (1) detailed course plan provided; (2) general course information provided; (3) conference tutorial/workshop outside of postsecondary environment; (4) rationale/arguments for teaching about ethical AI; (5) argument for teaching AI ethics in a particular way; (6) integrated ethical AI content (ethical AI content integrated with technical teaching); (7) stand-alone ethical AI content (course specifically about ethics); (8) content specific ethics (e.g., data ethics); and (9) description of course activity.

Course/ Curriculum Content. Reviewers will select from the following options: (1) fairness, accountability, and transparency (FAccT) combined concept; (2) fairness; (3) accountability; (4) transparency; (5) algorithmic bias; (6) privacy; (7) equity; (8) trust; (9) social responsibility; (10) general ethics/ethical thinking/ethical concepts; (11) safety; (12) security; (13) AI harms; (14) governance or regulation; (15) bias; (16) explainability; and (17) other.

Educational Setting. Reviewers will choose from the following educational settings: (1) undergraduate setting; (2) graduate setting; (3) computer science/engineering in general; (4) non-computer science; (5) other; and (6) not specified.

Educational Strategies. Possible educational strategies include: (1) case study; (2) real-world example; (3) fictional example; (4) debate, (5) role play; (6) lecture; (7) class discussions/reflections; (8) small group discussions/reflections; (9) peer-led discussions; (10) individual reflections; (11) discussion posts; (12) simulations; (13) group collaborations; (14) games; (15) situated learning; (16) media example(s); (17) crash courses; (18) compare/contrast ethical guidelines; (19) hands-on activity/applied AI; and (20) other.

Learning Outcomes Evaluation Method. Learning outcomes evaluation response options include: (1) survey; (2) observation; (3) assignment; (4) interview with students; (5) student reflections; (6) focus groups; (7) other; (8) not reported; and (9) not applicable.

Learning Outcomes Analysis Method. Possible approaches to analyzing learning outcomes include: (1) thematic analysis; (2) content analysis; (3) discourse analysis; (4) descriptive statistics; (5) other; and (6) none.

Reported Learning Outcomes. Potential reported learning outcomes include: (1) critical thinking skills (oral); (2) critical thinking skills (written); (3) ethical thinking; (4) empathy; (5) knowledge of ethical principles; (6) ability to evaluate the social impact of systems; (7) raising awareness of ethical concerns; (8) other; and (9) not reported.

Outcome Direction of Change. If researchers report on the direction of change for study outcomes, this will be charted as follows: (1) increase; (2) decrease; (3) mixed changes; (4) no change; (5) other; (6) not reported; and (7) not applicable.

### Step 5: Collating, summarizing, and reporting the results

Building on the data charting phase, the team will summarize the data according to each of the data charting elements. We will report on which elements have received the most and the least attention in this field of study, including specific examples of articles to illustrate what each data charting element represents. Following this, we will summarize recommended next steps to improve instructional design for teaching AI ethics in postsecondary contexts.

## Discussion

Results will include an up-to-date synthesis of the current state of AI ethics education in postsecondary curricula, evaluated teaching strategies, and potential outcomes associated with AI ethics education. This synthesis will include a discussion on the disciplinary gaps between the humanities and AI, informed by relevant research on this topic [42]. This review will inform future research, policy development, and teaching practices, offering valuable insights for educators, policymakers, and researchers working towards responsible AI integration. Findings will contribute to enhanced understandings of the complexities of AI ethics education and have the potential to shape the ways trainees in multiple disciplines learn about the ethical dimensions of AI in practice.

## Supporting information

S1 Table Teaching Ethical AI S1 Table 1 Sample Search Strategy. (DOCX)

S1 File PRISMA-P-SystRev-checklist Teaching Ethical AI. (DOCX)

S2 File PRISMA-ScR-TeachingAIEthics. (DOCX)
