# Supplementary material for: Teaching postsecondary students about the ethics of artificial intelligence: A scoping review protocol
Source: PLoS One. 2025 Jul 28;20(7):e0329020. doi: 10.1371/journal.pone.0329020 (PMC12303318; doi:10.1371/journal.pone.0329020)
Supplement: S1 Table — (DOCX) [file pone.0329020.s001.docx]

Full Search Strategy: PubMed

**PubMed (includes Medline), 23 June 2023**

|  | Search string | Results |
| --- | --- | --- |
| #1 | "Education"[MeSH Terms:noexp] OR "Curriculum"[MeSH Terms:noexp] OR "Education, Professional"[MeSH Terms] OR "Teaching"[MeSH Terms:noexp] OR "Schools, Health Occupations"[MeSH Terms] OR "Universities"[Mesh] OR "education"[MeSH Subheading] OR "Information Literacy"[Mesh] OR "Computer Literacy"[Mesh] OR teach*[tiab] OR instruct*[tiab] OR tutor*[tiab] OR educat*[tiab] OR pedagog*[tiab] OR paedagog*[tiab] OR curriculum*[tiab] OR course[tiab] OR courses[tiab] OR coursework[tiab] OR workshop*[tiab] OR "co op"[tiab] OR "information literacy"[tiab] OR "computer literacy"[tiab] OR universit*[tiab] OR college*[tiab] OR postsecondary[tiab] OR "post secondary"[tiab] OR "higher education"[tiab] OR undergraduate*[tiab] OR teach*[ta] OR learn*[ta] OR train*[ta] OR instruct*[ta] OR educat*[ta] OR pedagog*[ta] OR paedagog*[ta] OR universit*[ta] OR college*[ta] OR postsecondary[ta] OR "post secondary"[ta] OR "higher education"[ta] | 2,456,784 |
| #2 | "Ethics"[Mesh Terms] OR "ethics"[Mesh Subheading] OR "Morals"[Mesh Terms] OR "Ethicists"[Mesh Terms] OR "Human Rights"[MeSH Terms:noexp] OR "Civil Rights"[MeSH Terms:noexp] OR "Privacy"[Mesh Terms] OR "Confidentiality"[Mesh Terms] OR "Disclosure"[MeSH Terms:noexp] OR "Truth Disclosure"[MeSH Terms:noexp] OR "Duty to Warn"[Mesh Terms] OR "Bias"[MeSH Terms:noexp] OR "Social Discrimination"[Mesh Terms] OR "Prejudice"[Mesh Terms:noexp] OR "Racism"[Mesh Terms] OR "Sexism"[Mesh Terms] OR "Homophobia"[Mesh Terms] OR "Xenophobia"[Mesh Terms] OR "Sexual Harassment"[Mesh Terms] OR "Harassment, Non-Sexual"[Mesh Terms] OR "Cultural Competency"[Mesh Terms] OR "Paternalism"[Mesh Terms] OR "Beneficence"[Mesh Terms] OR ethic*[tiab] OR moral*[tiab] OR human right*[tiab] OR civil right*[tiab] OR harm[tiab] OR harms[tiab] OR privacy[tiab] OR confidentiality[tiab] OR consent[tiab] OR "right to erasure"[tiab] OR "right to be forgotten"[tiab] OR "right to information"[tiab] OR "data protection"[tiab] OR equality[tiab] OR equity[tiab] OR diversity[tiab] OR inclusivity[tiab] OR inclusiveness[tiab] OR fairness[tiab] OR bias[tiab] OR biases[tiab] OR biased[tiab] OR discrimination[tiab] OR prejudic*[tiab] OR racist*[tiab] OR racism*[tiab] OR sexist*[tiab] OR sexism[tiab] OR homophob*[tiab] or homo phob*[tiab] OR transphob*[tiab] OR trans phob*[tiab] OR harassment*[tiab] OR cultural compentenc*[tiab] OR paternalism[tiab] OR decoloniz*[tiab] OR de coloniz*[tiab] OR decolonis*[tiab] OR de colonis*[tiab] OR accountability[tiab] OR verifiability[tiab] OR replicability[tiab] OR transparent[tiab] OR transparency[tiab] OR explainability[tiab] OR trustworthy*[tiab] OR "environmental responsibility"[tiab] OR "environmentally responsible"[tiab] OR security[tiab] OR "open source"[tiab] OR professionalism[tiab] OR "professional responsibility"[tiab] OR integrity[tiab] OR "human control"[tiab] | 1,658,564 |
| #3 | "Artificial Intelligence"[MeSH Terms:noexp] OR "Machine Learning"[MeSH Terms] OR "Data Mining"[MeSH Terms:noexp] OR "Neural networks, Computer"[MeSH Terms] OR "Algorithms"[MeSH Terms:noexp] OR "Computing Methodologies"[MeSH Terms:noexp] OR "Computer Systems"[MeSH Terms] OR "Artificial intelligence"[tiab] OR "Machine learning"[tiab] OR "deep learning"[tiab] OR artificial neural network*[tiab] OR large language model*[tiab] OR alexnet[tiab] OR generative adversarial network*[tiab] OR biggan[tiab] OR stylegan[tiab] OR resnets[tiab] OR alphago[tiab] OR "Generative Pretrained Transformer"[tiab] OR gpt[tiab] OR chatgpt[tiab] OR "Bidirectional Encoder Representations from Transformers"[tiab] OR bert[tiab] OR "self supervised learning"[tiab] OR alphafold[tiab] OR "Dall e"[tiab] OR "github copilot"[tiab] OR "Stable diffusion"[tiab] OR llama[tiab] OR alpaca[tiab] OR bard[tiab] OR "palm 2"[tiab] OR computer*[ti] OR computing[ti] OR algorithm*[ti] OR "data mining"[ti] | 741,757 |
| #4 | #1 AND #2 AND #3 | 8,291 |
